# An audio-semantic multimodal model for automatic obstructive sleep Apnea-Hypopnea Syndrome classification via multi-feature analysis of snoring sounds

**DOI:** 10.3389/fnins.2024.1336307

**Published:** 2024-05-10

**Authors:** Xihe Qiu, Chenghao Wang, Bin Li, Huijie Tong, Xiaoyu Tan, Long Yang, Jing Tao, Jingjing Huang

**Affiliations:** ^1^School of Electronic and Electrical Engineering, Shanghai University of Engineering Science, Shanghai, China; ^2^INF Technology (Shanghai) Co., Ltd., Shanghai, China; ^3^Department of Otolaryngology, Shenzhen Second People's Hospital, Shenzhen, China; ^4^Eye & ENT Hospital of Fudan University, Shanghai, China

**Keywords:** obstructive sleep Apnea-Hypopnea Syndrome, snoring sounds, semantic information, PubMedBERT, multimodal model

## Abstract

**Introduction:**

Obstructive Sleep Apnea-Hypopnea Syndrome (OSAHS) is a common sleep-related breathing disorder that significantly impacts the daily lives of patients. Currently, the diagnosis of OSAHS relies on various physiological signal monitoring devices, requiring a comprehensive Polysomnography (PSG). However, this invasive diagnostic method faces challenges such as data fluctuation and high costs. To address these challenges, we propose a novel data-driven Audio-Semantic Multi-Modal model for OSAHS severity classification (i.e., ASMM-OSA) based on patient snoring sound characteristics.

**Methods:**

In light of the correlation between the acoustic attributes of a patient's snoring patterns and their episodes of breathing disorders, we utilize the patient's sleep audio recordings as an initial screening modality. We analyze the audio features of snoring sounds during the night for subjects suspected of having OSAHS. Audio features were augmented via PubMedBERT to enrich their diversity and detail and subsequently classified for OSAHS severity using XGBoost based on the number of sleep apnea events.

**Results:**

Experimental results using the OSAHS dataset from a collaborative university hospital demonstrate that our ASMM-OSA audio-semantic multimodal model achieves a diagnostic level in automatically identifying sleep apnea events and classifying the four-class severity (normal, mild, moderate, and severe) of OSAHS.

**Discussion:**

Our proposed model promises new perspectives for non-invasive OSAHS diagnosis, potentially reducing costs and enhancing patient quality of life.

## 1 Introduction

Obstructive Sleep Apnea-Hypopnea Syndrome (OSAHS) is a prevalent sleep-breathing disorder worldwide, significantly impacting patients' health and quality of life. However, the gold standard of OSAHS diagnosis typically involves complex and time-consuming processes, relying on resource-intensive methods of Polysomnography (PSG). This invasive method limits its applicability in large-scale screening and may potentially result in delays in patient intervention and treatment, thereby exacerbating health risks. Considering that OSAHS patients experience recurrent partial or complete upper airway blockages during sleep, leading to breathing pauses or reduced airflow, these sleep apnea events can result in a range of clinical consequences, including daytime sleepiness, fatigue, hypertension, diabetes, lipid abnormalities, cognitive impairments, cardiovascular events, and even mortality (Franklin and Lindberg, 2015). Therefore, developing a simple, precise, and cost-efficient approach to automatically diagnose the severity of OSAHS is crucial. In recent years, snoring has garnered significant attention as an early symptom of OSAHS. Snoring, which results from the vibration of upper airway structures, is observed in nearly 80% of OSAHS patients. Analyzing snoring audio collected from OSAHS patients can provide essential information about sleep apnea events.

Current OSAHS screening approaches rely on physiological signals extracted from PSG, such as electroencephalography (EEG) and electrooculography (EOG), to classify the severity of OSAHS in patients. However, obtaining these signals requires patients to connect multiple signal recorders, which complicates the extraction process, increases costs, and fails to precisely differentiate between different degrees of OSAHS patients (Emoto et al., 2007; Azarbarzin and Moussavi, 2010; Franklin and Lindberg, 2015; Qian et al., 2016, 2019). In addition to employing invasive PSG, some researchers (Chatburn and Mireles-Cabodevila, 2011; Ma et al., 2015, 2024; Albornoz et al., 2017; Likitha et al., 2017; Winursito et al., 2018) utilize the conspicuous correlation between patients' snoring audio and the severity of OSAHS. Hou et al. (2021) proposed an innovative approach for estimating the Sleep Apnea Hypopnea Index (AHI) by analyzing snore sounds using the Equivalent Rectangular Bandwidth (ERB) correlation dimension, demonstrating a potential non-invasive method for assessing the risk of sleep apnea. These studies have achieved a certain degree of success in classifying OSAHS by analyzing patients' sleep audio features. However, challenges persist in accurately categorizing patients into distinct classifications, such as normal, mild, moderate, and severe.

**Figure 1:**
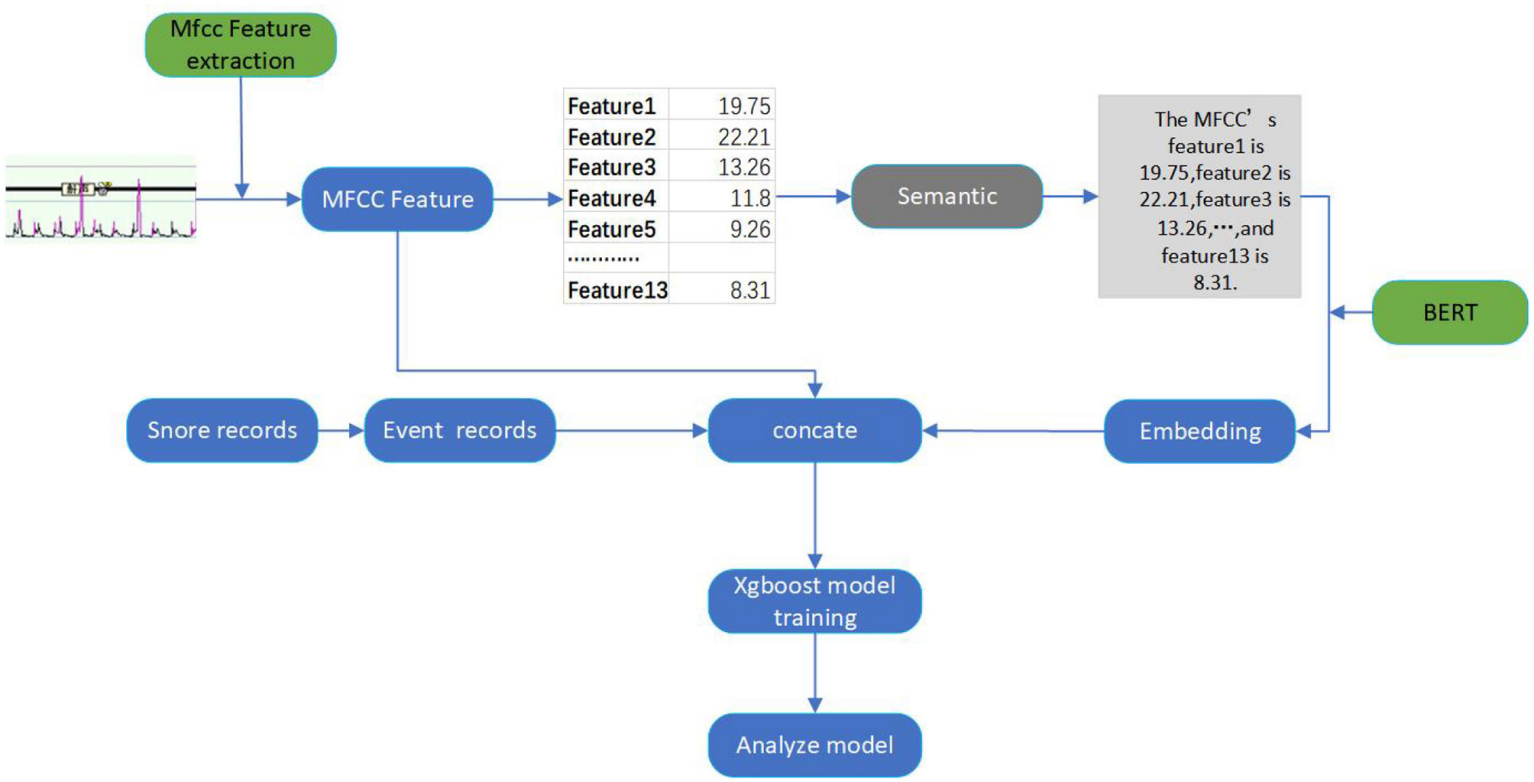
The overall framework of our proposed audio-semantic multimodal model ASMM-OSA.

To address these challenges, we have introduced a multi-feature analysis-based audio-semantic multimodal model for assessing the severity of OSAHS in patients. We concentrate on analyzing sleep audio recordings from subjects suspected of having OSAHS, with the goal of both streamlining the screening process and diagnosing the severity of OSAHS. As shown in Figure 1, we automatically segment patients' audio data and extract Mel-frequency Cepstral Coefficients (MFCC) as audio features. Concurrently, we employ the PubMedBERT language model (Tinn et al., 2023) to transform these audio features into text features, capturing correlations among MFCC dimensions and thereby enhancing feature discriminability. Subsequently, we concatenate the audio features and text features to form the final sleep audio features for suspected patients. We utilize an XGBoost (Chen and Guestrin, 2016) to calculate the total number of sleep apnea events and compute the AHI score (Malhotra et al., 2021), which is used to classify the severity of OSAHS in patients. In conclusion, we concatenate the audio and text features through multimodal data fusion, leveraging both audio and text features, improving XGBoost's robustness for the automatic classification of OSAHS severity. This approach offers insights into early OSAHS diagnosis and treatment. Specifically, the main contributions of our work are as follows:

- We present ASMM-OSA, a data-driven multimodal model for analyzing sleep audio data in individuals susceptible to OSAHS. Our objective is to automatically detect sleep apnea events and assess their severity. Through the fusion of audio and text features via feature concatenation, we successfully integrate these modalities. Employing XGBoost for preliminary classification, our model effectively distinguishes various sleep apnea event types within audio segments. This approach enhances audio feature diversity, thereby improving OSAHS classification reliability. Additionally, our model enhances feature representation and achieves superior classification accuracy.
- By leveraging a pre-trained language model PubMedBERT, we incorporate patient-specific semantic information, including vital signs, pertinent medical history, and other relevant data, to aid in overnight snoring audio diagnosis. Our results demonstrate superior performance, demonstrating the effects of integrating semantic prior knowledge in enhancing classification accuracy.
- We extensively evaluate our ASMM-OSA using a clinical dataset from a collaborative university hospital. Our model outperforms baseline methods, achieving a state-of-the-art diagnosis accuracy of 77.6% in identifying sleep apnea events, offering a rapid and effective automatic tool for early diagnosis.

The rest of the paper is organized as follows. Section 2 reviews related work on snoring features and semantic information extraction. Section 3 introduces the proposed framework, and Section 4 describes detailed experimental results and ablation studies. Section 5 concludes the paper.

## 2 Related work

### 2.1 Snoring feature extraction

Due to the strong correlation between snoring and OSAHS, prior research typically concentrates on analyzing patients' snoring and subsequently determining whether they have OSAHS. Emoto et al. (2007) utilized second-order autoregressive models to characterize snoring sounds and analyze the severity of OSAHS. Azarbarzin and Moussavi (2010) proposed an automatic unsupervised snoring detection algorithm that used two microphones, one on the trachea and one in the surrounding environment, to capture respiratory sound signals from patients. The vertical box (V-Box) algorithm was then employed to identify sound segments as either snoring or non-snoring. Qian et al. (2019) used machine listening techniques to identify the obstruction and vibration positions in the upper airway of subjects, analyzing their snoring data and employing a naive Bayes model as a classifier. Similarly, Qian et al. (2016) introduced a novel feature set based on wavelet transform and a support vector machine classifier to differentiate Velum, Oropharynx lateral wall, Tongue base, and Epiglottis (VOTE) snoring data detected by in-sleep endoscopy, distinguishing between OSAHS patients and primary snorers. However, these studies are confined to focusing on the analysis of snoring features to ascertain whether patients snore or have OSAHS. Despite their success, they still face difficulty accurately categorizing OSAHS severity into multiple classes (normal, mild, moderate, and severe cases), limiting its potential usage in assisting medical diagnoses.

The analysis of snoring characteristics is fundamentally a part of audio analysis. Currently, Chatburn and Mireles-Cabodevila (2011) and Likitha et al. (2017) have successfully utilized acoustic features to analyze speakers' emotions, achieving excellent performance. Building on this foundation, in recent years, researchers have employed MFCC for acoustic feature analysis to classify different types of snoring. Albornoz et al. (2017) utilized MFCC to extract snoring features and employed a support vector machine (SVM) for snoring type classification. Ma et al. (2015) detected snoring candidates using V-Box and then extracted MFCC features from each candidate to classify snoring or non-snoring using the K-Harmonic Mean clustering algorithm. Sun et al. (2020) used MFCC to obtain snoring features and applied support vector machines for snoring localization. However, most of these methods either do not classify the types of snoring accurately or do not focus on OSAHS severity assessment, which is crucial for effective patient treatment.

### 2.2 Semantic information extraction

Pre-trained language models have been widely demonstrated as extremely effective. These language models are typically pre-trained on large text datasets and can be fine-tuned in different domains to extract distinctive features, exhibiting exceptional performance across various tasks.

In the medication domain, Shin et al. (2020) proposed BioMegatron, an improvement on BERT, to understand biomedical language and context. Fang and Wang (2022), Chen et al. (2021), and Tian and Zhang (2021) used PubMedBERT for COVID-19 literature classification and annotation. Almeida et al. (2022) utilized PubMedBERT's contextual embeddings to enhance document retrieval and question-answering tasks. Bevan and Hodgskiss (2021) employed BERT to learn feature representations of chemical entities. Portelli et al. (2021) evaluated and selected SpanBERT and PubMedBERT for medical text recognition. Liu et al. (2022) introduced MetBERT for predicting metastatic cancer from clinical records. Lin et al. (2021) proposed EntityBERT, a BERT-based model, to explore the clinical domain using a masked strategy. Miao et al. (2021) and collaborators utilized PubMedBERT for CID entity relation classification through Text-CNN fine-tuning. Danilov et al. (2021) applied PubMedBERT to classify short scientific texts and demonstrated better performance compared to other models. Zhang et al. (2021) used models like BioBERT and PubMedBERT for generating answers in QA tasks, with PubMedBERT showing good performance but not matching the BioBERT-MNLI-SQuAD model fine-tuned on external datasets, potentially due to differences in pre-training corpora. Rao (2022) tested the capabilities of BERT models in microbiology text mining and compared their applicability. Gupta et al. (2023) proposed an automated report generation method combining a visual transformer and PubMedBERT. Shen et al. (2022) and Mullin et al. (2023) employed various BERT models to analyze electronic health records of Alzheimer's disease patients and study the impact of lifestyle on the disease. Wang et al. (2021) introduced Cross-contrast BERT for obtaining semantic information in biomedical tasks. In the medical field, pre-trained language models have been widely used to extract text features from medical records, including patient medical histories and symptom descriptions. This text information aids doctors in disease diagnosis, prediction, and treatment decisions. Although previous research has utilized BERT models (Devlin et al., 2018; Qi et al., 2023) and extensions such as PubMedBERT and BioBERT, these models have not been applied to extracting text information from patients' snoring data, which has primarily focused on electronic health records.

## 3 Method

Figure 1 demonstrates the overall framework of our proposed method, comprising audio feature extraction, semantic feature conversion, feature concatenation, and classification.

### 3.1 Audio feature extraction

For each patient's sleep audio, we utilize the Mel Frequency Cepstral Coefficients (MFCC) to extract audio features. MFCC, a widely used method in audio signal processing, excels at capturing spectral sound characteristics. This process involves multiple steps, including preprocessing, Fast Fourier transform (FFT), Mel filter banks, logarithmic operations, Discrete Cosine Transform (DCT), as well as dynamic feature extraction. The specific steps of this process are as follows.

#### 3.1.1 Fast Fourier Transform

After performing data framing and windowing preprocessing steps, we apply the Fast Fourier Transform (FFT) to map the obtained time-domain signal into the frequency domain. This transformation is then converted into a power spectrum, which facilitates the subsequent transformation to the Mel scale.


$$\begin{array}{l} X\left(k\right)=\overset{N-1}{\underset{n=0}{\sum }}x\left(n\right)\cdot e^{-j\frac{2\pi }{N}nk} \end{array}$$

Where *x*(*n*) represents the input audio signal, *N* is the frame size, and *k* is the frequency index.

#### 3.1.2 Mel filter banks

The Mel filter bank is employed to transform the linear spectrum into the Mel spectrum.


$$\begin{array}{l} {\text{S}}_{\text{m}}=\overset{\text{K}}{\underset{\text{k}=1}{\sum }}|\text{X}\left(\text{k}\right){|}^{2}\cdot {\text{H}}_{\text{m}}\left(\text{k}\right) \end{array}$$

Where *X*(*k*) represents the spectral values of the audio frame,where *k* ranges from 1 to *K*(often *K* is half the number of FFT points), representing different frequency components. while *H*_*m*_(*k*) corresponds to the response of the *m*-th Mel filter.

#### 3.1.3 Logarithmic operations

After obtaining the energy values for each Mel filter as described above, a logarithm is taken to prepare for cepstral analysis.


$$\begin{array}{l} M_{m}=\log\left(S_{m}\right) \end{array}$$

#### 3.1.4 Discrete Cosine Transform

The obtained Mel energy values for each filter are then subjected to Discrete Cosine Transform (DCT) to obtain the final Mel-Frequency Cepstral Coefficients (MFCCs).


$$\begin{array}{l} C_{l}=\overset{M}{\underset{m=1}{\sum }}M_{m}\cdot \cos\left[\frac{\pi l}{M}\left(m-0.5\right)\right] \end{array}$$

Following these steps, we obtain MFCC feature vectors for each minute, including 13-dimensional static coefficients that reflect the energy distribution and spectral characteristics of snoring sounds. These MFCC feature vectors can then be utilized for subsequent data processing.

### 3.2 Semantic feature transformation

We employ a pre-trained PubMedBERT model to transform the MFCC features. We encode the MFCC features for each minute into a sentence-like format and input them into the PubMedBERT model to obtain semantic information. PubMedBERT is a model trained on a large-scale collection of medical literature data, endowed with robust semantic representation capabilities, enabling it to effectively capture specific features and knowledge within the medical domain.

We utilize the notation *C*_1_, *C*_2_, …, *C*_*t*_ to denote the MFCC feature vector for each minute, where *Ct* represents the MFCC features at the *t* minute. These MFCC features are structured into a sentence-like format:


$$\begin{array}{l} S_{\text{input}}=\left[C_{1},C_{2},\ldots ,C_{t}\right] \end{array}$$

Where *S*_input_ is the obtained sentence, the encoding format is shown in Figure 1. We then input this sentence into the PubMedBERT model to get semantic information:


$$\begin{array}{l} S_{\text{output}}=\mathrm{PubMedBERT}\left(S_{\text{input}}\right) \end{array}$$

*S*_output_ represents the semantic feature obtained by PubMedBERT.

### 3.3 Processing snoring event records

We extracted snoring event records for each minute from the text files corresponding to the audio data. This yielded a 480-dimensional event record dataset, where each entry indicated the presence or absence of a snoring event for a specific minute. We further converted these records into binary labels: 1 represented the sleep apnea event lasting 30 s or more within the minute, while 0 denoted the normal sleep event. This transformation facilitated the integration of snoring event information into subsequent analyses.


$$\begin{array}{l} \text{E}=\left[{\text{e}}_{1},{\text{e}}_{2},\ldots ,{\text{e}}_{\text{T}}\right] \end{array}$$

Where e_T_ represents the event records for each minute, with 1 indicating the sleep apnea event and 0 indicating the normal sleep event. These records form a 480-dimensional dataset.

### 3.4 Feature concatenation

We then concatenate the obtained semantic feature with the sleep apnea event records. The audio data is 480-dimensional, and the semantic feature we obtained after transformation is also 480-dimensional, aligning with the dimensionality of the event records. By concatenating the MFCC features, semantic features, and event records along their respective dimensions, we built a comprehensive feature. The feature serves as the input for the classification model, which is used for training and prediction purposes.


$$\begin{array}{l} F_{t}=\left[s_{t},e_{t},C_{t}\right] \end{array}$$

Where *s*_*t*_ represents the semantic feature of the *t* minute, *e*_*t*_ represents the event record of the *t* minute, *C*_*t*_ is a 480-dimensional vector, and *F*_*t*_ represents the comprehensive feature of the *t* minute. This comprehensive feature includes semantic features, event records, and audio features.

### 3.5 Classification model

XGBoost (Chen and Guestrin, 2016), based on gradient boosting trees, incrementally integrates multiple decision tree models to enhance predictive performance. This iterative approach allows XGBoost to extract knowledge from multiple relatively weak learners, gradually approaching the true complexity of the problem. In the diagnosis of OSAHS, a multitude of features are at play, and the capabilities of XGBoost empower us to more effectively capture these complex relationships, consequently enhancing diagnostic accuracy.

The loss function of XGBoost consists of two parts: the regularization term and the data fitting term. Specifically, the loss function of XGBoost can be expressed as:


$$\begin{array}{l} \text{loss}=\overset{n}{\underset{i=1}{\sum }}\ell \left(y_{i},{ŷ}_{i}\right)+\overset{K}{\underset{k=1}{\sum }}\Omega \left(f_{k}\right) \end{array}$$

Where,*n* is the training sample size,*y*_*i*_ is the ground-truth label,ŷ_*i*_ is the predicted label,*K* is the number of trees,*f*_*k*_ represents the *k*-th tree, ℓ(*y*_*i*_, ŷ_*i*_) is the data fitting term, and Ω(*f*_*k*_) is the regularization term.

We adopt the XGBoost to construct a classification model aimed at determining the presence of sleep apnea events on a per-minute basis. The model takes as input the comprehensive feature data obtained through prior steps. By training the model, we are able to predict sleep apnea events for each minute, consequently enabling the computation of the total count of such events throughout the entire night. This predictive capacity of the XGBoost model plays a pivotal role in diagnosing the severity of obstructive sleep apnea, offering insights into the patient's sleep-related respiratory patterns.

Based on the total count of overnight sleep apnea events, we can assess the severity of a patient's sleep apnea syndrome. We calculate the Apnea-Hypopnea Index (AHI) for each individual, computed as the count of events with occurrences during the entire night's sleep divided by the duration of the sleep.


$$\begin{array}{l} AHI=\frac{N}{t} \end{array}$$

Where *N* denotes the count of sleep apnea events during a patient's night of sleep, and *t* signifies the duration of that night's sleep in hours. Subsequently, the patient's severity of OSAHS is categorized into four levels using the AHI index, including simple snoring (*AHI* < 5), mild (5 ≤ *AHI* < 15), moderate (15 ≤ *AHI* < 30), and severe (*AHI*≥30) (Marti-Soler et al., 2016).

Additionally, we employ metrics such as Mean Squared Error(MSE), Mean Absolute Error(MAE), precision, recall, F1 score, and Area Under the ROC Curve (AUC) to assess the model's performance, ensuring its accuracy and reliability. The AUC measures the area under the ROC curve, with a range from 0 to 1. The larger the AUC value, the better the performance of the classifier.

The definitions of these six metrics are as follows:


$$\begin{array}{l} \text{MAE}=\frac{1}{\text{n}}\overset{\text{n}}{\underset{\text{i}=1}{\sum }}|{\text{y}}_{\text{i}}-{\hat{\text{y}}}_{\text{i}}| \end{array}$$

$$\begin{array}{l} \mathrm{MSE}=\frac{1}{\text{n}}\overset{\text{n}}{\underset{\text{i}=1}{\sum }}\left({\text{y}}_{\text{i}}-{\hat{\text{y}}}_{\text{i}}\right) \end{array}$$

$$\begin{array}{l} {\text{R}}_{-}^{2}\text{Score}=1-\frac{\underset{\text{i}}{\sum }{\left({\text{y}}_{\text{i}}-{\hat{\text{y}}}_{\text{i}}\right)}^{2}}{\underset{\text{i}}{\sum }{\left(\bar{\text{y}}-{\hat{\text{y}}}_{\text{i}}\right)}^{2}} \end{array}$$

where: *y*_*i*_ denotes the ground-truth label, ŷ_*i*_ denotes the prediction and ȳ denotes the mean value.


$$\begin{array}{l} \text{Precision}=\frac{TP}{TP+FP} \end{array}$$

$$\begin{array}{l} \text{Recall}=\frac{TP}{TP+FN} \end{array}$$

$$\begin{array}{l} F1_{-}\text{Score}=\frac{2\times \text{Precision}\times \text{Recall}}{\text{Precision}+\text{Recall}} \end{array}$$

## 4 Experiments and results

### 4.1 Data description and processing

The dataset was collected from the Eye & Ent Hospital of Fudan University consisting of 250 patients. The dataset includes patients' audios and corresponding OSAHS severity and event labels, labeled by three clinical experts. The data acquisition and processing followed these steps: first, we recorded 8 h of sleep audio for each patient at a sampling rate of 8 kHz. Subsequently, the sleep recordings were divided into hourly segments, generating eight-hour audio clips, each containing sleep state and snoring information for its corresponding hour. For each hourly audio clip, a 13-dimensional MFCC feature extraction was applied, which effectively captures audio spectral features. Then, the MFCC features are standardized to have a mean of 0 and a variance of 1, enhancing the stability and effectiveness of the model. The dataset is split into 80% for training and 20% for testing. Table 1 is a depiction of our dataset, including the number of patients in the training and test sets, the number of patients with different types of snoring, the number of males and females, and the age distribution.

**Table 1:** Description of the dataset.

**Dataset**	**Sample size**	**Normal**	**Mild**	**Moderate**	**Severe**	**Age < 40**	**Age ≥40**	**Male**	**Female**
Train	200	26	45	44	82	67	133	178	22
Test	50	11	10	8	21	21	29	40	10

To obtain event labels, the sleep apnea events and their durations were annotated in the respective text based on the patient's overnight sleep pattern. Event determination relied on whether the event duration exceeded 30 s, thus deciding the presence of a sleep apnea event per minute. Combining each patient's MFCC values with their corresponding event labels resulted in a 480-dimensional dataset. This dataset was further combined with the previously acquired semantic information, creating a comprehensive dataset containing semantic information, MFCC features, and event labels for training and testing experiments.

### 4.2 Hyper-parameters settings and model evaluation

During training, the model's hyperparameters (such as learning rate, number of trees, maximum depth, etc.) are adjusted to optimize the model's performance. Table 2 shows our hyperparameter settings. We utilized cross-validation to choose the best combination of hyperparameters and avoid overfitting.

**Table 2:** Optimal parameters for the classification model.

**Parameter name**	**Parameter meaning**	**Optimal parameters**
Leaning rate	Learning rate	0.3
Max depth	Maximum depth of the tree	9
Subsample	Subsampling rate of the sample	0.6
Reg lambda	L2 regularization	10
Reg alpha	L1 regularization	0.1
Colsample bytree	Subsampling rate of the feature	0.5
Gamma	Control the splitting process of the tree	0.2

### 4.3 Main results

#### 4.3.1 Performance in identifying sleep apnea events

We selected 50 patients as the test set and divided them into 10 groups, with each group containing five patients, conducting a total of 10 experiments. Through training the dataset with the XGBoost model, using MFCC values as features, we predicted the occurrence and type of sleep apnea events per minute and compared them with the ground-truth sleep apnea events, thus assessing the predictive performance of the model. The results presented in Table 3 showed that our model achieved superior performance in identifying sleep apnea events, with an average accuracy of 77.6%, and an average AUC of 0.709.

**Table 3:** Sleep apnea event classification results for 10 groups of patients.

**Patient group number**	**Accuracy**	**Precision**	**Recall**	**F1-score**	**AUC**	**Specificity**
01	0.963	0.850	0.975	0.908	0.968	0.961
02	0.967	0.965	0.960	0.963	0.966	0.972
03	0.675	0.506	0.692	0.584	0.679	0.667
04	0.825	0.861	0.766	0.810	0.824	0.882
05	0.700	0.296	0.301	0.298	0.554	0.658
06	0.659	0.523	0.662	0.584	0.660	0.885
07	0.829	0.544	0.587	0.565	0.736	0.853
08	0.716	0.417	0.311	0.357	0.582	0.881
09	0.681	0.490	0.248	0.329	0.564	0.809
10	0.742	0.192	0.300	0.234	0.554	0.972
Average	0.776	0.564	0.580	0.563	0.709	0.837

**Figure 2:**
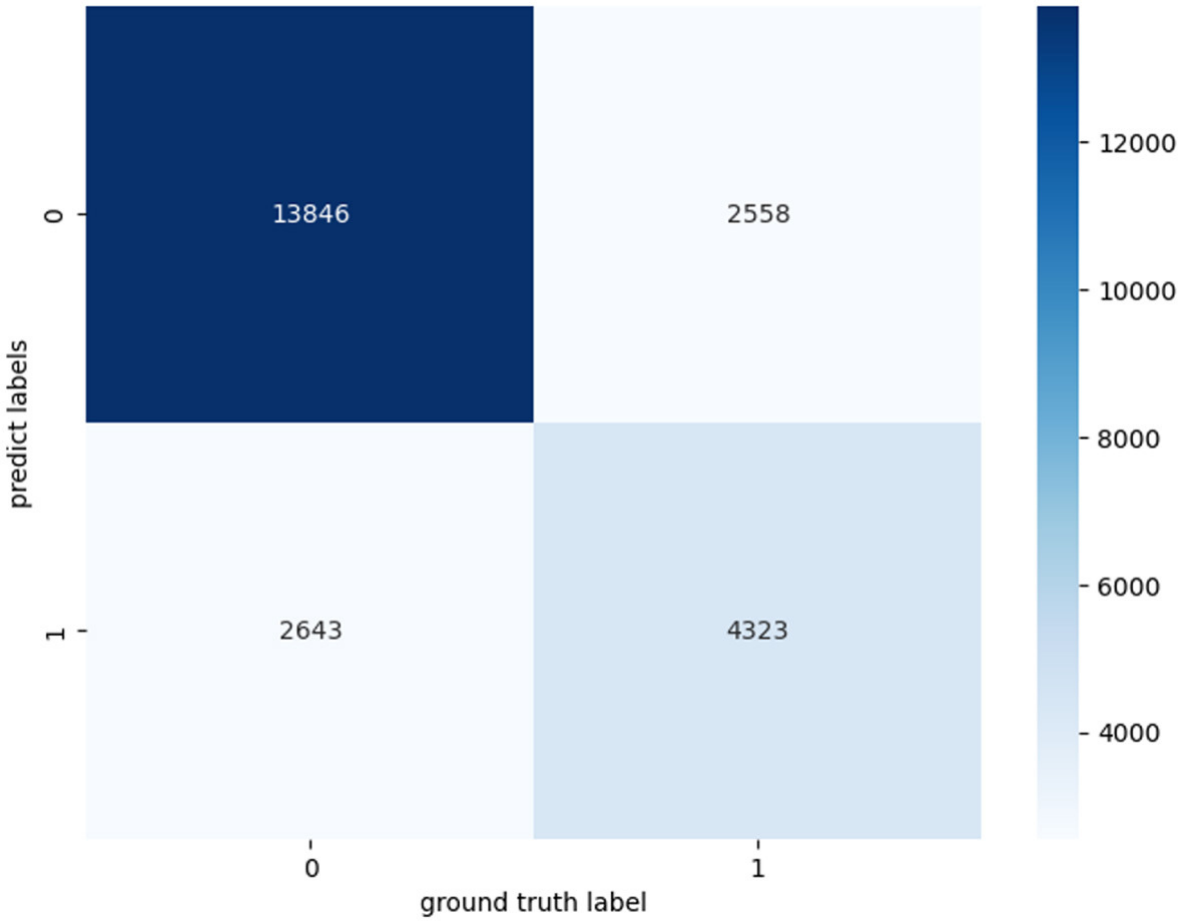
Confusion matrices for apnea event classification.

In addition, we have further evaluated our proposed model using a confusion matrix (Figure 2). A confusion matrix is a fundamental tool for assessing the performance of a binary classification model. TP represents the cases where our model correctly identified positive instances, meaning it accurately detected the condition we were interested in, such as a medical condition or an event. TN stands for the cases where the model correctly recognized negative instances, indicating that it correctly ruled out the presence of the condition or event. In our study, we analyzed 23,370 samples, These samples originate from audio snippets of participants' entire night's sleep, each audio sample representing whether an apnea event occurred. Following the preprocessing of these audios, the total number of samples amounted to 23,370. The essential characteristic of these samples is the MFCC per minute. Label 0 represents a normal sleep event, while 1 indicates the occurrence of an apnea event. And the confusion matrix revealed 12,869 TP cases and 4,643 TN cases. These results demonstrate the model's efficacy in accurately discerning the presence of sleep apnea events. The abundance of TP cases underscores its accuracy in identifying positive instances, while the significant TN count signifies its proficiency in correctly excluding negative instances.

**Figure 3:**
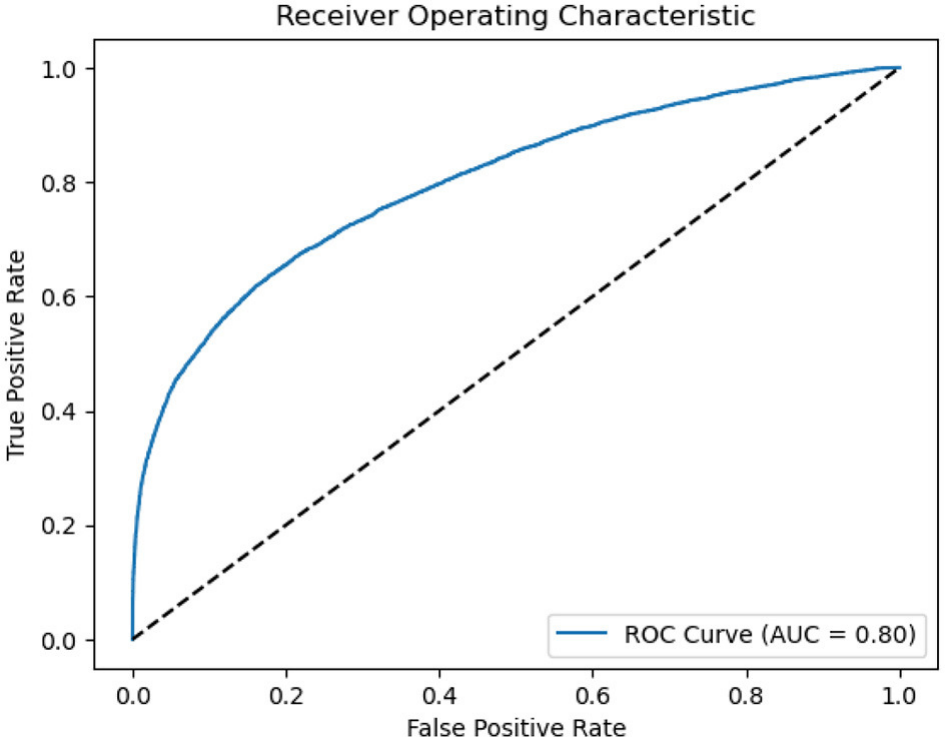
ROC of ASMM-OSA. The horizontal axis quantifies the proportion of healthy cases incorrectly identified as diseased, while the vertical axis quantifies the proportion of diseased cases correctly identified.

As presented in Figure 3, we also employed The Receiver Operating Characteristic (ROC) curves for model evaluation. The ROC curve is a graphical tool that visually assesses a model's binary classification performance. It plots the True Positive Rate (TPR), also known as Sensitivity, against the False Positive Rate (FPR), reflecting the model's accuracy in classifying positive instances while minimizing negative. A higher AUC value close to 1 indicates the model's superior capacity to differentiate between positive and negative instances. In our study, an AUC of 0.8 indicates a strong discriminative ability in distinguishing sleep apnea events.

#### 4.3.2 Important parameter analysis

**Figure 4:**
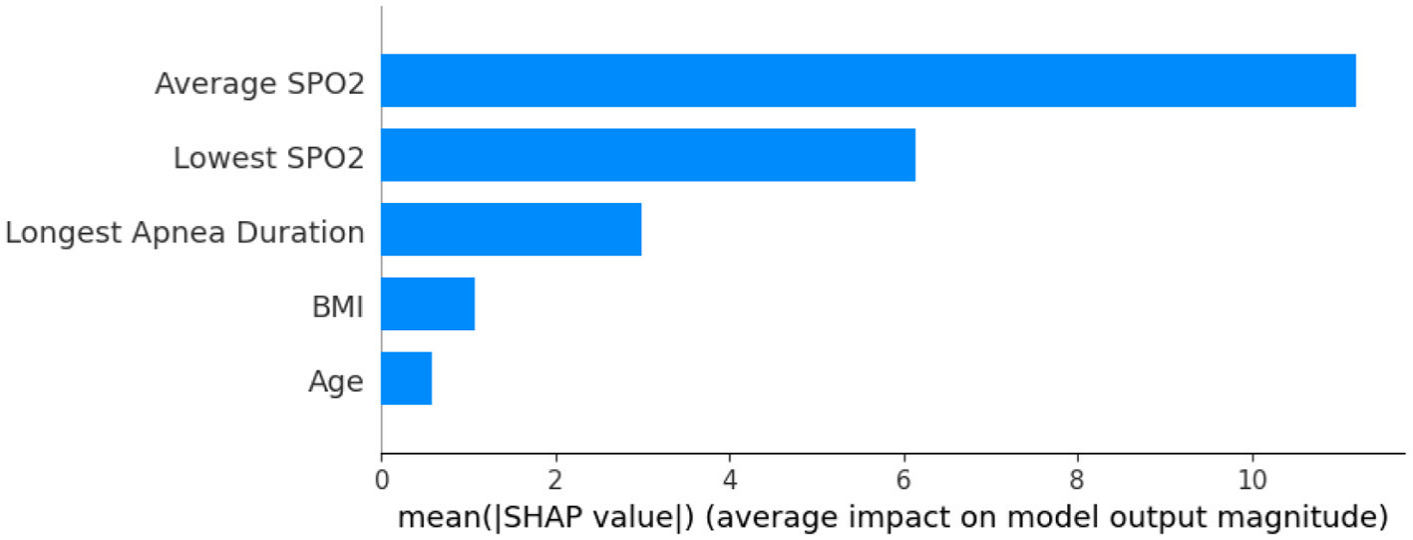
Feature importance ranking.

In this paper, we use the SHAP model to perform an interpretive analysis of the XGBoost model's output. The importance ranking of the features of the SHAP is shown in Figure 4, where the most important feature in the AHI predict process is average SPO2, In addition to average SPO2, Lowest SPO2, and Longest Apnea Duration are also an important indicator for AHI predict.

**Figure 5:**
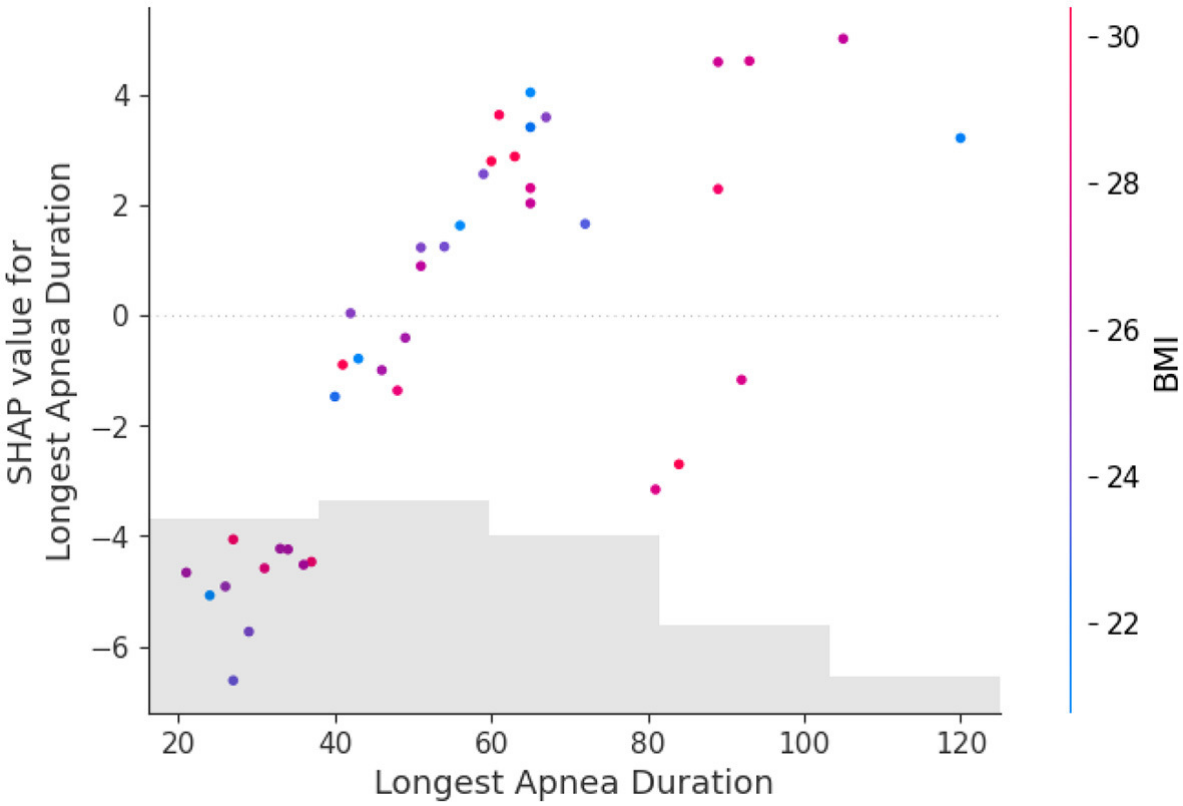
The dependence between longest apnea duration and BMI. The gray histogram represents the sample distribution of the Longest Apnea Duration, while points of different colors depict the BMI of different samples.

Figure 5 shows the feature dependence plots of “Longest Apnea Duration”. The scatter plot reveals the contributions of “Longest Apnea Duration” and “BMI” to the model prediction. The gray histogram represents the sample distribution of “Longest Apnea Duration”, while points of different colors depict the distribution of SHAP values for sample features with various BMI. The larger the SHAP value, the greater the positive contribution of the corresponding feature value in the model. The color represents the numerical values of BMI, with the color bar displaying the BMI value ranges corresponding to different colors. The figure indicates a positive correlation between “Longest Apnea Duration” and SHAP values, implying that as the duration of apnea prolongs, the corresponding SHAP values increase, suggesting a higher dependence on this feature. Meanwhile, the distribution of BMI appears more scattered, which indicates the influence of BMI on “Longest Apnea Duration” is relatively independent rather than linearly correlated, underscoring the complexity of the model's prediction.

### 4.4 Comparative experimental results of different models

#### 4.4.1 Compare to baselines

In our experiments, we compared our model with other models from related studies, and the results are shown in Table 4, which indicates that our metrics outperform other snoring classification prediction models in several assessment metrics. The chosen baselines are as follows, implemented according to the details specified within each baseline:

- Limin et al. (2019) extracted MFCC and used the Gaussian mixture model to model and classify snoring sound all night, and then estimated the AHI index of the subjects.
- Zhao et al. (2021) extracted the formants of snoring sound and compared them with the personalized threshold to describe the difference between OSAHS patients and simple snorers, and estimated the simulated AHI of subjects.
- Shen et al. (2020) applied the Long Short Term Memory (LSTM) to explore deeply representative features from MFCC, LPC, and the integration of LPCC and MFCC.
- Ding et al. (2023) detected apnea hypopnea-related snoring sound based on analysis of the Mel-spectrogram.

**Table 4:** Experimental results for the differentiation of sleep apnea events using the baseline classification approach.

**Model**	**Accuracy**	**Precision**	**Recall**	**F1-score**
GMMs (Limin et al., 2019)	0.769	0.550	0.501	0.496
Random Forest (Zhao et al., 2021)	0.709	0.491	0.507	0.533
LSTM (Shen et al., 2020)	0.680	0.509	0.511	0.559
VGG19-LSTM (Ding et al., 2023)	0.758	0.554	0.568	0.524
ASMM-OSA	**0.776**	**0.564**	**0.580**	**0.563**

The best performance is indicated in bold.

#### 4.4.2 Classify the severity of OSAHS

We utilize a test set consisting of 50 patients, calculating the AHI score for each patient and determining their corresponding severity level. These results are then compared to the severity levels derived from the ground truth derived from PSG. We label 0, 1, 2, and 3 to represent the different degrees of OSAHS severity: 0 denotes normal, 1 signifies mild, 2 indicates moderate, and 3 represents severe.

Table 5 presents the label distribution of patients with different severity level labels and the predictive accuracy of the four-class classification. The overall predictive accuracy of the four classes is 58%. It is noted that our model exhibits superior performance in categorizing patients with moderate and severe conditions. In practical situations, prioritizing the classification of moderate and severe cases is crucial, given their potential need for urgent hospital treatment. Consequently, the diagnostic outcomes facilitate swift and precise identification of moderate and severe patients, enabling expedited and effective interventions by healthcare professionals.

**Table 5:** The number of people with each symptom in PSG and test results.

	**0**	**1**	**2**	**3**	**All**
PSG	14	18	6	12	50
Accuracy	50%	50%	66.7%	75%	58%

Table 6 shows our model accurately estimates the actual Apnea-Hypopnea Index (AHI) for most subjects, especially for patients 1–7 and 9. The prediction discrepancies observed in patients 8 and 10 can be attributed to patient diversity and the first-night effect, which refers to variations in sleep patterns during an individual's first exposure to a sleep study environment. Additionally, the size of the dataset may have impacted the model's generalizability. Despite the challenges of data variability and size, Table 4 demonstrates that our approach outperforms the baseline on the same dataset, highlighting the effectiveness of our proposed model.

**Table 6:** The comparison of OSAHS degree of our method compared with the gold standard PSG results in 10 random patients.

**Patient number**	**AHI (PSG)**	**AHI (test)**	**Label (PSG)**	**Label (test)**	**AUC**
01	10.5	5.5	1	1	0.968
02	5.5	7	1	1	0.966
03	68.7	48.625	3	3	0.679
04	3.2	3.625	0	0	0.824
05	3.8	2.250	0	0	0.554
06	7.1	5.375	1	1	0.660
07	14.2	18	1	2	0.736
08	46	15.625	0	2	0.582
09	2.0	2.125	0	0	0.564
10	4.9	11.875	0	1	0.554

#### 4.4.3 Ablation study

We further conducted ablations concentrating on evaluating the impact of semantic information features on the classification performance of the ASMM-OSA. We designed two components, with one utilizing MFCC as the feature and the other concatenating semantic features as the final feature representation. The results are presented in Table 7. From the experimental results, it can be observed that after incorporating PubMedBERT, the average accuracy increased to 0.776, compared to 0.746 without utilizing PubMedBERT. Furthermore, there was a discernible improvement in precision and ASMM-OSA demonstrated superior precision in classifying positive samples when PubMedBERT was employed.

**Table 7:** The results of the ablation experiment sleep apnea event classification in 10 groups of patients.

**Group no**.	**Acc**	**Acc (w/o.)**	**Prec**	**Prec (w/o.)**	**Recall**	**Recall (w/o.)**	**F1**	**F1 (w/o.)**	**AUC**	**AUC (w/o.)**
01	0.963	0.892	0.850	0.647	0.975	0.902	0.908	0.753	0.968	0.896
02	0.967	0.908	0.965	0.899	0.960	0.894	0.963	0.897	0.966	0.906
03	0.675	0.649	0.506	0.48	0.692	0.790	0.584	0.598	0.679	0.685
04	0.825	0.826	0.861	0.849	0.766	0.784	0.810	0.815	0.824	0.825
05	0.700	0.679	0.296	0.307	0.301	0.408	0.298	0.351	0.554	0.580
06	0.659	0.664	0.523	0.526	0.662	0.709	0.584	0.604	0.660	0.674
07	0.829	0.812	0.544	0.502	0.587	0.686	0.565	0.580	0.736	0.764
08	0.716	0.708	0.417	0.424	0.311	0.440	0.357	0.432	0.582	0.619
09	0.681	0.672	0.490	0.447	0.248	0.343	0.329	0.398	0.564	0.584
10	0.742	0.654	0.192	0.174	0.300	0.465	0.234	0.248	0.554	0.561
Average	0.776	0.746	0.564	0.526	0.580	0.642	0.563	0.568	0.709	0.709

Due to PubMedBERT being pre-trained on a large-scale biomedical literature dataset, ASMM-OSA has acquired enhanced semantic feature-capturing capabilities, particularly excelling in capturing medical domain-specific features. Comparatively, incorporating PubMedBERT significantly improves the performance of ASMM-OSA in classifying the severity of OSAHS patients when contrasted with not introducing this language model.

## 5 Conclusion

In this paper, we introduce an audio-semantic multimodal model for the classification of OSAHS severity (i.e., ASMM-OSA). We integrate patients' sleep audio features with semantic features and employ XGBoost to classify sleep apnea events, thereby calculating the patient's AHI score to assess the severity of OSAHS. Experimental results demonstrate the enhancement in classification performance achieved by incorporating semantic information, highlighting the superior performance of ASMM-OSA in classifying sleep apnea events. This approach provides a robust tool for precisely diagnosing sleep-related disorders. In the future, we will conduct a thorough analysis of performance variations among different patient groups. We will investigate aspects such as feature selection, model fine-tuning, and other enhancements to further improve model performance and its generalization capabilities.

## Data availability statement

The raw data supporting the conclusions of this article will be made available by the authors, without undue reservation.

## Ethics statement

The studies involving humans were approved by the Ethics Committee of the Eye & ENT Hospital of Fudan University. The studies were conducted in accordance with the local legislation and institutional requirements. The participants provided their written informed consent to participate in this study.

## Author contributions

XQ: Methodology, Supervision, Writing—original draft. CW: Software, Writing—original draft. BL: Validation, Writing—review & editing. HT: Writing—review & editing. XT: Writing—review & editing. LY: Writing—review & editing. JT: Data curation, Writing—review & editing. JH: Data curation, Resources, Supervision, Writing—review & editing.
